# Cytogenotoxic Effects of Fluopyram in *Allium cepa* Root Cells Involving Microtubule Disruption

**DOI:** 10.1002/tox.70096

**Published:** 2026-04-12

**Authors:** Carlos Filipe Camilo‐Cotrim, Natanael Alves da Silva, Gabriela Gomes Lima, Igor Rodrigues Vasconcelos, Luciana Souza Ondei, Elisa Flávia Luiz Cardoso Bailão, Antônio Sérgio Nakao de Aguiar, Marcelino Benvindo‐Souza, Leonardo Luiz Borges, Virgínia Damin, Luciane Madureira Almeida

**Affiliations:** ^1^ Laboratório de Biologia, Universidade Estadual de Goiás Unidade Universitária de Porangatu Porangatu Goiás Brazil; ^2^ Laboratório de Biotecnologia Universidade Estadual de Goiás Anápolis Goiás Brazil; ^3^ Laboratório de Biogeografia e Ecologia Aquática Universidade Estadual de Goiás Anápolis‐Go Brazil; ^4^ Laboratório de Novos Materiais Universidade Evangélica de Goiás Anápolis Goiás Brazil; ^5^ Grupo de Química Teórica e Estrutural de Anápolis Universidade Estadual de Goiás Anápolis Goiás Brazil; ^6^ Escola de Ciências Médicas e da Vida Pontifícia Universidade Católica de Goiás Goiânia Goiás Brazil; ^7^ Herbil, Universidade Federal de Goiás Escola de Agronomia ‐ Campus Samambaia Goiânia Goiás Brazil

**Keywords:** aberrations, abnormalities, aneugenicity, biomonitoring, ecotoxicology

## Abstract

Fluopyram is a next‐generation fungicide widely used in agriculture; however, its persistence in soils and mobility toward aquatic systems have raised concerns regarding potential ecotoxicological risks. This study evaluated the cytogenotoxic effects of a commercial fluopyram‐based pesticide formulation on *Allium cepa* root meristem cells. Bulbs were exposed to four concentrations based on agricultural application rates: three times the recommended field dose (3 × FD; 5.00 mg mL^−1^), the field dose (FD; 1.67 mg mL^−1^), half the field dose (0.5 × FD; 0.835 mg mL^−1^), and one‐tenth the field dose (0.1 × FD; 0.17 mg mL^−1^). Endpoints assessed included lipid peroxidation, cell viability, cytogenetic alterations, and DNA damage. No evidence of oxidative stress was detected. Nevertheless, the fluopyram‐based formulation induced significant chromosomal aberrations and nuclear abnormalities at all tested concentrations, indicating pronounced genotoxic potential. The comet assay revealed a low frequency of DNA strand breaks, suggesting an aneugenic mode of action likely associated with mitotic spindle disruption rather than clastogenic damage. Mitotic analysis showed an accumulation of cells in prophase, reinforcing the interpretation of chromosome missegregation. *In silico* docking analyses further demonstrated strong interactions between fluopyram ions and tubulin, providing mechanistic support for spindle‐associated aneugenicity. Overall, these findings highlight the cytogenotoxic risks associated with fluopyram‐based pesticides and reinforce the importance of environmental monitoring to protect nontarget organisms.

## 1 Introduction

Pesticides are integral to modern agriculture, ensuring crop protection and enhancing productivity. However, their intensive use has raised growing concerns about environmental and human health risks [1]. Fluopyram is a new‐generation broad‐spectrum fungicide that also functions as a nematicide, seed treatment, and post‐harvest preservative. Its biological activity stems from the inhibition of succinate dehydrogenase (SDH), a key enzyme in the mitochondrial respiratory chain. For this reason, fluopyram belongs to the class of succinate dehydrogenase inhibitors (SDHIs) [2].

The environmental behavior of fluopyram raises particular concerns due to its high persistence in soils (half‐life > 30 days) and mobility, which increase the risk of leaching into surface and groundwater systems [3, 4, 5]. Risk assessments using the Groundwater Ubiquity Score (GUS) and Goss method classify fluopyram as having high contamination potential [6, 7]. In addition, its degradation may yield transformation products with unknown or potentially toxic properties [8].

In nontarget organisms, fluopyram has been associated with oxidative stress [9], shifts in soil microbial communities [10], and potential carcinogenicity in vertebrates [11, 12]. As an SDHI (succinate dehydrogenase inhibitors), fluopyram can interfere with mitochondrial electron transport, contributing to its broad toxicity profile [13]. Despite these concerns, its genotoxic effects, particularly in plant systems, remain underexplored.

The *Allium cepa* test system is a widely accepted model for genotoxicity and cytotoxicity assessment due to its sensitivity to chromosomal abnormalities, low cost, and correlation with other bioassays [14, 15]. Its enzymatic pathways are also comparable to those in mammalian cells, allowing the detection of cellular and physiological responses relevant to eukaryotic systems [16]. Several studies have successfully applied cytogenotoxic and genotoxic endpoints, including chromosomal aberrations and DNA damage, to evaluate pesticide effects using the *A. cepa* bioassay, as summarized in a comprehensive review [14].

In this study, we evaluated the cytogenotoxic potential of a commercial fluopyram‐based pesticide formulation in *A. cepa* root meristem cells exposed to concentrations based on agricultural application dose. The assessed parameters included lipid peroxidation, cell viability, chromosomal abnormalities, and DNA damage. This work contributes to the understanding of fluopyram's biological impact and supports the need for improved environmental risk assessment of SDHI fungicides.

## 2 Methodology

### 2.1 Material

The pesticide Verango Prime (Fluopyram—500 g L^−1^, Bayer) was used in this study. Tested concentrations were based on field recommendations for pre‐emergence application in soybean crops (0.5 L ha^−1^ of product diluted in 150 L ha^−1^ of water). Four concentrations were evaluated: 5.00 mg mL^−1^ (3× field dose: 3× FD), 1.67 mg mL^−1^ (field dose: FD), 0.835 mg mL^−1^ (50% of field dose: 0.5× FD), and 0.17 mg mL^−1^ (10% of field dose: 0.1× FD). All concentrations were prepared with distilled water, accounting for the active ingredient in the formulation. Table 1 describes the chemical properties of the fluopyram active ingredient.

**TABLE 1:** Chemical properties of fluopyram, active ingredient of Verango Prime.

Common name	CAS name	Formula	Chemical structure	Use
Fluopyram	*N*‐(2‐(3‐chloro‐5‐(trifluoromethyl)‐2‐pyridinyl)ethyl)‐2‐(trifluoromethyl)benzamide	C_16_H_11_ClF_6_N_2_O	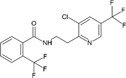	Fungicide, nematicide

### 2.2 Experimental Procedure

The experiment was conducted four times, each addressing specific endpoints. In the first experiment, roots were collected to assess lipid peroxidation by quantifying malondialdehyde (MDA), a marker of oxidative stress and cellular damage. The second and third experiments collected roots for cell viability evaluation using Evans blue and 2,3,5‐triphenyltetrazolium chloride (TTC) staining. The fourth experiment collected roots for cytological slide preparation to analyze the mitotic index (MI), chromosomal aberrations (CA), nuclear abnormalities (NA), and DNA damage by the comet assay.

Five bulbs were used per pesticide concentration, as well as for negative and positive controls. Each experiment condition was performed in triplicate, totaling 360 bulbs (Ø = 31.03 ± 0.63 mm, 19.79 ± 1.36 g) of the yellow variety of *Allium cepa* (2n = 2× = 16 chromosomes), obtained from the Centro Estadual de Abastecimento (CEASA) of Anápolis, Goiás, Brazil.

Bulbs were initially cleaned by removing external layers and excess dry roots, while maintaining intact root primordia. They were then placed in distilled water for 5 days for cytotoxicity assays and for 48 h for genotoxicity assays, at 23°C in a BOD incubator, with daily water renewed after 24 h. After root development, bulbs were exposed to pesticide concentrations and controls for 48 h, with pesticide solutions changed after 24 h. Following exposure, roots were collected, washed, and fixed in Carnoy's solution for 24 h, and subsequently stored in 70% ethanol.

Zinc sulfate heptahydrate (6 mg mL^−1^) was used as a positive control for cytotoxicity assessment [17, 18], while methyl methanesulfonate (10 mg mL^−1^) was used as a positive control for genotoxicity evaluation [19]. Distilled water was used as the negative control. Both positive controls were applied under the same experimental conditions and exposure time as the treatments.

### 2.3 Lipid Peroxidation

Following exposure, 50 mg of root tissue was collected from each bulb to assess lipid peroxidation by quantifying malondialdehyde (MDA) using the TBARS assay [20]. Roots were homogenized in 200 μL of Tris–HCl 0.1 M (pH 8) using a micro‐homogenizer (Marconi MA1102). Thiobarbituric acid (TBA; 0.03 M) was added, and samples were incubated at 90°C for 40 min. After cooling, n‐butanol was added, followed by centrifuging at 2800 ×*g* (Thermo Scientific Heraeus Megafuge 16R) for 10 min. Absorbance of the supernatant was measured at 535 nm in a 96‐well plate reader (SpectraMax Paradigm Multi‐mode).

### 2.4 Cell Viability

Cell death was assessed by Evans blue staining [21]. Roots were immersed in 1.5 mL of 2.6 mM Evans blue solution at 25°C for 15 min under agitation, washed, and 10 mm of the root tips excised for analysis. The dye was extracted using 50% methanol + 35 mM SDS at 50°C for 1 h, and absorbance was measured at 600 nm (SpectraMax Paradigm).

Metabolically active cells were evaluated by TTC staining [22, 23]. Root tips were incubated in 1 mL of 15 mM TTC solution at 35°C for 15 min in the dark. Dye extraction was carried out with 95% ethanol, and absorbance was read at 490 nm.

### 2.5 Cytogenetic Analysis

Cytogenetic analysis followed a modified method by Fernandes et al. [24, 25]. After exposure, roots were collected and fixed in Carnoy's solution (ethanol: acetic acid, 3:1) for 24 h, then stored in 70% ethanol until slide preparation. On the day of preparation, roots were washed and hydrolyzed in 1 M HCl at 60°C for 10 min, followed by staining with Schiff's reagent for 45 min in the dark at 23°C.

Meristematic regions were excised, placed on microscope slides, and dissociated in 2% acetic carmine. The material was spread using a razor blade, covered with a coverslip, briefly heated, and immersed in liquid nitrogen to remove the coverslips. Permanent slides were mounted using Entellan.

For each bulb, two slides were prepared, and 500 cells were analyzed per slide, totaling 1000 cells per bulb (5000 cells per treatment across three replicates = 15 000 cells). Microscopic analysis was conducted using an optical microscope (Primo Star, Carl Zeiss) and photomicrographs captured with a camera module (Axiocan 105 color, Carl Zeiss). Genotoxic effects were evaluated in interphase and mitotic cells, classifying chromosomal aberrations and nuclear abnormalities according to Leme and Marin‐Morales [26].

### 2.6 Comet Assay

The comet assay was performed to evaluate genotoxicity according to the modified protocols of Gichner et al. [27] and Türkoğlu [28]. After treatment, root tips were incubated in 60 μL of cold tris buffer (400 mM, pH 7.5), macerated, and mixed with 240 μL of 0.1% low‐melting‐point agarose (40°C). The suspension was spread onto slides pre‐coated with 1.5% agarose, covered with coverslips, and solidified at 4°C for 3 min.

Slides were kept on ice for 1 h, washed, and immersed in alkaline electrophoresis buffer (EDTA and NaOH) for 20 min. Electrophoresis was conducted in the dark for 30 min at 25 V and 300 mA. Slides were then neutralized three times with 0.4 M Tris buffer (pH 7.5) for 5 min, rinsed with distilled water, and fixed with absolute ethanol. DNA was stained with 100 μL of Diamond fluorescent dye and examined under a fluorescence microscope (Axio Imager A2, Carl Zeiss AG, Germany) equipped with excitation filters at 510–560 nm, emission at 590 nm, and a 20× objective lens. A total of 100 nucleoids per sample were analyzed by a single examiner using CometScore 1.5 software. The parameters evaluated were %DNA in the tail, tail length, and Olive tail moment.

### 2.7 Docking Analysis

Following evidence of potential interaction between fluopyram and tubulin, the target PDB ID: 7Z2P (Protein Data Bank, https://www.rcsb.org/) was selected for docking analysis to construct interaction models [29]. Many conformations of fluopyram were simulated (10 best poses were finally selected) in complex with the structure of the biological target specified and the fluopyram to verify the fit favorably into the ligand binding site of the biomolecule. The target protein was downloaded, and the water molecules were removed. After identifying the active site, the dimensions adopted for the docking were a grid box of 10 Å. The genetic algorithm was employed. The program GOLD Suite 5.7.0 (Mark Thompson and Planaria Software LLC) was used to generate the docking models. The 2D interaction maps were produced using Poseview [30], and the 3D images were built with PyMOL Molecular Graphics System, Version 3.1.3 software (https://www.pymol.org/). Redocking was performed using a structure in which the target protein and its ligand were co‐crystallized to validate the models produced with our selected molecules. Default values were employed for all other parameters, and structures were submitted to 10 genetic algorithm runs using the CHEMPLP fitness function.

### 2.8 Pharmacophore Modeling

To create shared features and pharmacophore models for selective ligands of the tubulin, we employed LigandScout v4.5 (https://docs.inteligand.com/ligandscout/ accessed on July 29, 2025) [31]. LigandScout identified combinations of shared pharmacophoric features, including hydrogen‐bond donors (HBDs), hydrogen‐bond acceptors (HBAs), positively and negatively charged groups, hydrophobic (HyPho) regions, and aromatic rings (ARs) [32]. These unique pharmacophores were subsequently integrated into the alignment process to develop the Shared Feature Pharmacophore (SFP) model. A literature review was conducted using the databases in Binding DB (https://www.bindingdb.org/rwd/bind/index.jsp accessed on July 29, 2025) to identify compounds with tubulin antagonist activity. Five compounds with the lowest inhibitory concentration (IC_50_) values were included in a dataset used to obtain pharmacophore models.

### 2.9 Data Analysis

Data were analyzed using one‐way ANOVA to compare pesticide concentrations with the control group. Assumptions of absence of significant outliers, normality, and homogeneity of variances were tested to ensure the reliability of the outcomes. When assumptions were violated, data were transformed using the Box–Cox method. If the transformed data still did not meet the assumptions, the nonparametric Kruskal–Wallis test was applied. For cases where ANOVA assumptions were met, pairwise comparisons were conducted using Tukey's post hoc test. Statistical analyses were performed considering a significance level of *p* < 0.05.

R software, version 4.2.2 [33], and RStudio, version 2024.9.0 [34], were used for all statistical procedures. The statistical analysis employed the dplyr, MASS, rstatix, tidyverse, and vegan packages. nMDS plotting required the ggplot2 package. GraphPad Prism, version 9.0.0 for Windows (GraphPad Software, San Diego, CA, USA, www.graphpad.com), provided the other graphs. Adobe Illustrator, version 24.0.1, composed the figures.

## 3 Results

### 3.1 Oxidative Stress and Cell Viability

Oxidative stress, assessed by malondialdehyde (MDA) levels, differed between the positive control and all other groups (*F*_(5,12)_ = 229.68; *p* < 0.0001; *η*_*g*_^2^ = 0.99). Both the negative control and the tested concentrations of the fluopyram‐based pesticide showed similar values, indicating that none of the evaluated concentrations induced lipid peroxidation (Figure 1a).

**FIGURE 1:**
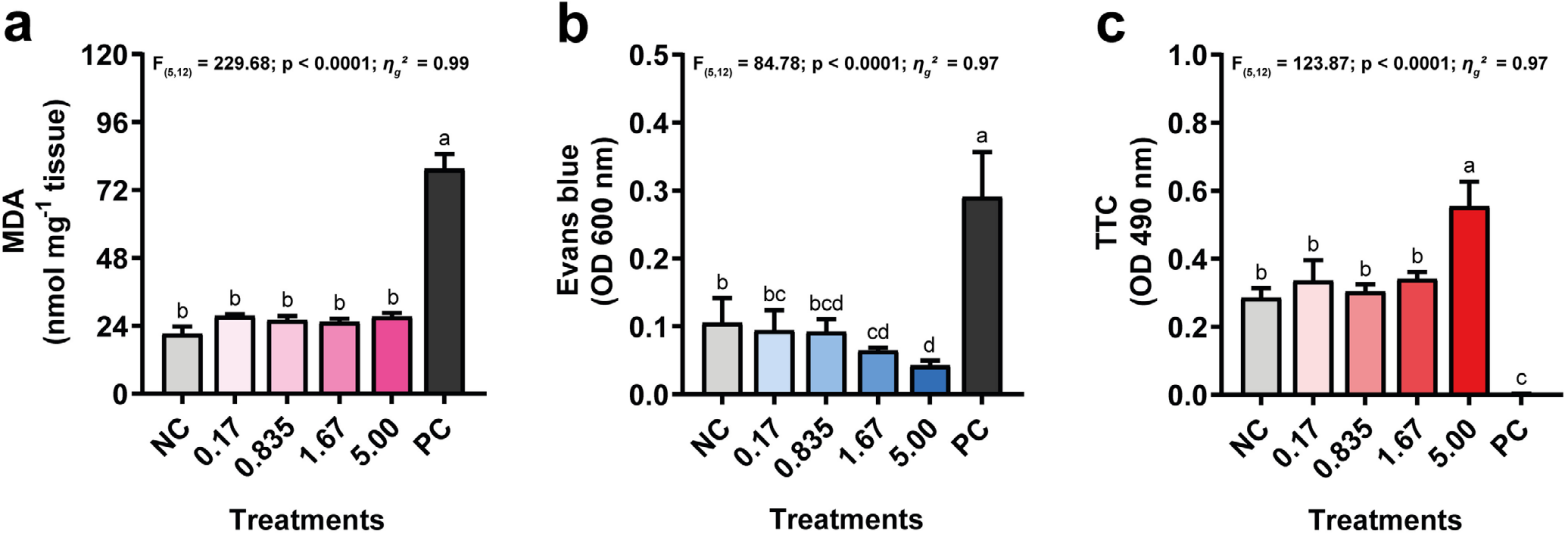
Cytotoxicity parameters in *Allium cepa* roots exposed for 48 h to different concentrations of a fluopyram‐based pesticide and controls. Bars represent mean ± standard deviation. (a) MDA values. (b) Optical density of Evans blue dye released after staining, indicating cell membrane integrity. (c) Optical density of formazan produced after TTC staining, indicating metabolic activity. MDA = Malondialdehyde; TTC = 2,3,5‐triphenyltetrazolium chloride; CN = negative control; PC = positive control. Different letters indicate significant differences among treatments according to Tukey's post hoc test (*p* < 0.05); bars sharing the same letter do not differ significantly.

Cell viability assessed by the Evans blue assays differed among treatments (*F*_(5,12)_ = 84.78; *p* < 0.0001; *η*_*g*_^*2*^ = 0.97). A significant reduction in absorbance was observed at the recommended field dose (1.67 mg mL^−1^) and at three times this concentration (5.00 mg mL^−1^) compared to the negative control (Figure 1b), indicating enhanced membrane integrity and increased cell viability at higher concentrations. Similarly, the TTC assay revealed significant variation among treatments (*F*_(5,12)_ = 123.87; *p* < 0.0001; *η*_*g*_^*2*^ = 0.97), with markedly higher absorbance at 5.00 mg mL^−1^ (Figure 1c), consistent with increased metabolic activity. Overall, these results indicate that higher concentrations of fluopyram‐based pesticide are associated with increased cellular activity.

### 3.2 Cytotoxic Effects and Cell Cycle Phases

Mitotic index analysis revealed differences among treatments (*F*_(5,12)_ = 12.37; *p* < 0.001; *η*_*g*_^*2*^ = 0.83; Figure 2a). An increase in the mitotic index was observed at 0.835 mg mL^−1^ compared to the negative control. The mitotic index inhibition/stimulation calculation applied in this study measured the relative difference between the mitotic index of the treatment and that of the control, normalized by the control's mitotic index. The resulting value was multiplied by −100, which reverses the sign; therefore, positive values indicate stimulation, whereas negative values indicate inhibition. Regarding mitotic index modulation, the concentrations of 0.835 and 1.67 mg mL^−1^ increased cell division by more than 20% relative to the negative control, indicating mitogenic effects during the exposure period (Figure 2b).

**FIGURE 2:**
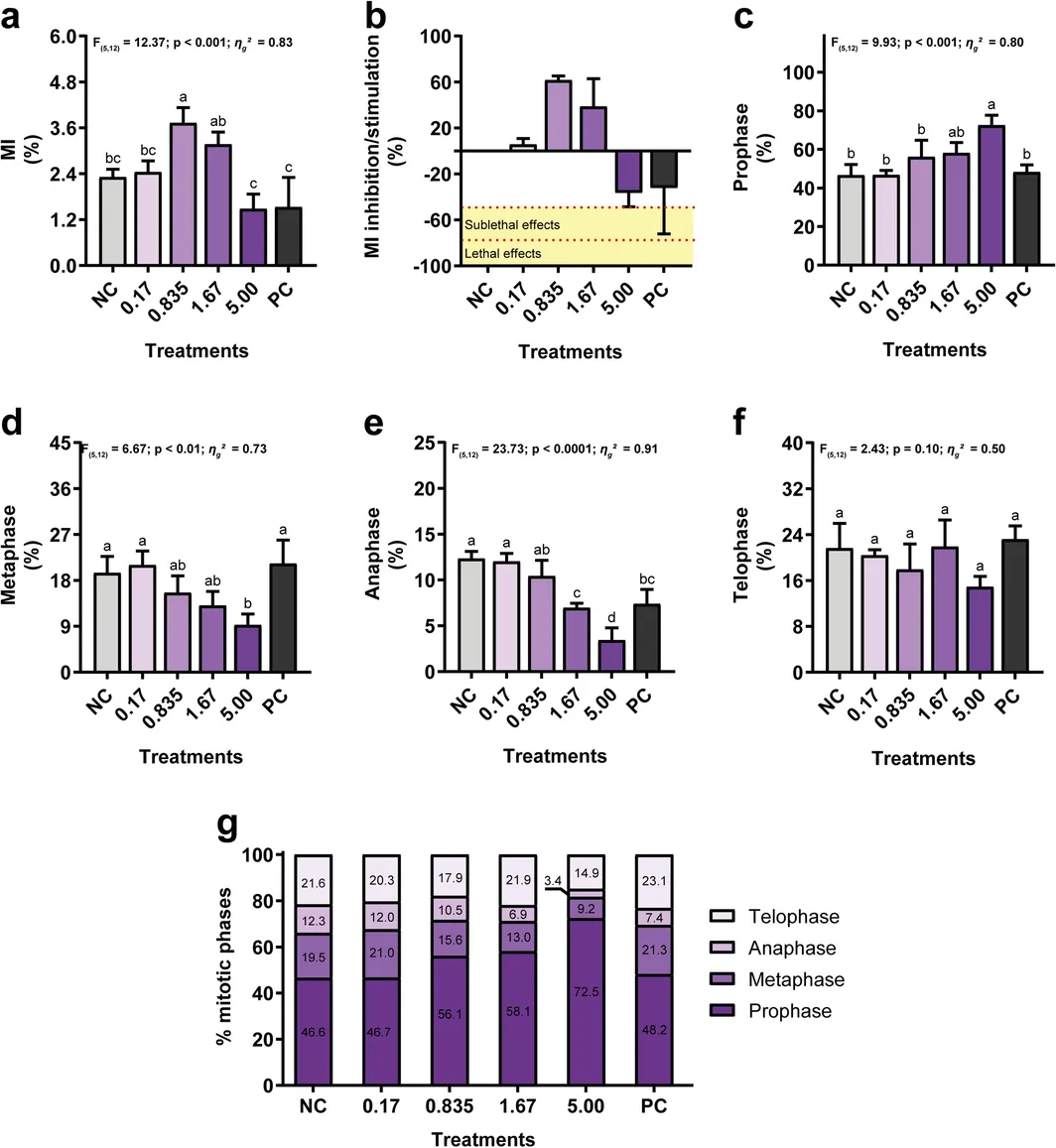
Mitotic and cell cycle parameters in *Allium cepa* roots exposed for 48 h to different concentrations of a fluopyram‐based pesticide and controls. Bars represent mean ± standard deviation. (a) Mitotic index (MI). (b) Percentage of MI inhibition or stimulation relative to the negative control (values below −50% indicate sublethal effects and below −78% lethal effects; positive values indicate stimulation). (c–f) Distribution of cells in prophase, metaphase, anaphase, and telophase, respectively. (g) % mitotic phases. Different letters indicate significant differences according to Tukey's post hoc test (*p* < 0.05); bars sharing the same letter do not differ significantly.

Assessment of mitotic phase distribution revealed alterations across treatment groups. In prophase (Figure 2c), a marked increase in the number of cells was detected at 5.00 mg mL^−1^ (*F*_(5,12)_ = 9.93; *p* < 0.001; *η*_*g*_^*2*^ = 0.80), indicating that fluopyram‐based pesticide exposure led to cell cycle arrest at the onset of mitosis. Conversely, the proportion of cells in metaphase (Figure 2d) declined significantly with increasing concentrations (*F*_(5,12)_ = 6.67; *p* < 0.01; *η*_*g*_^*2*^ = 0.73), with the greatest reduction observed at 5.00 mg mL^−1^. Anaphase frequencies (Figure 2e) were similarly affected (*F*_(5,12)_ = 23.73; *p* < 0.0001; *η*_*g*_^*2*^ = 0.91), with marked reductions at 1.67 and 5.00 mg mL^−1^, supporting evidence of impaired mitotic progression. In contrast, telophase (Figure 2f) was not significantly altered by the treatments (*F*_(5,12)_ = 2.43; *p* = 0.10; *η*_*g*_^*2*^ = 0.50), remaining relatively stable across concentrations. Overall, prophase was the most frequent stage, accounting for more than 45% of dividing cells in all treatments (Figure 2g). Collectively, these findings indicate that fluopyram‐based pesticide alters mitotic dynamics in a concentration‐dependent manner, promoting entry into mitosis while impairing progression through subsequent phases.

### 3.3 Genotoxicity Effects

Chromosomal aberration analysis showed significant differences among treatments (*F*_(5,12)_ = 7.01; *p* < 0.01; *η*_*g*_^*2*^ = 0.74). All concentrations of the fluopyram‐based pesticide exhibited higher frequencies of aberrations than the negative control (Figure 3a), with values comparable to those observed in the positive control. Similarly, nuclear abnormalities differed significantly among treatments (*F*_(5,12)_ = 4.17; *p* < 0.02; *η*_*g*_^*2*^ = 0.63), with elevated frequencies in all fluopyram‐based pesticide groups relative to the negative control (Figure 3b), and no significant differences from the positive control. Together, these results demonstrate the genotoxic potential of the fluopyram‐based formulation through the induction of chromosomal and nuclear alterations.

**FIGURE 3:**
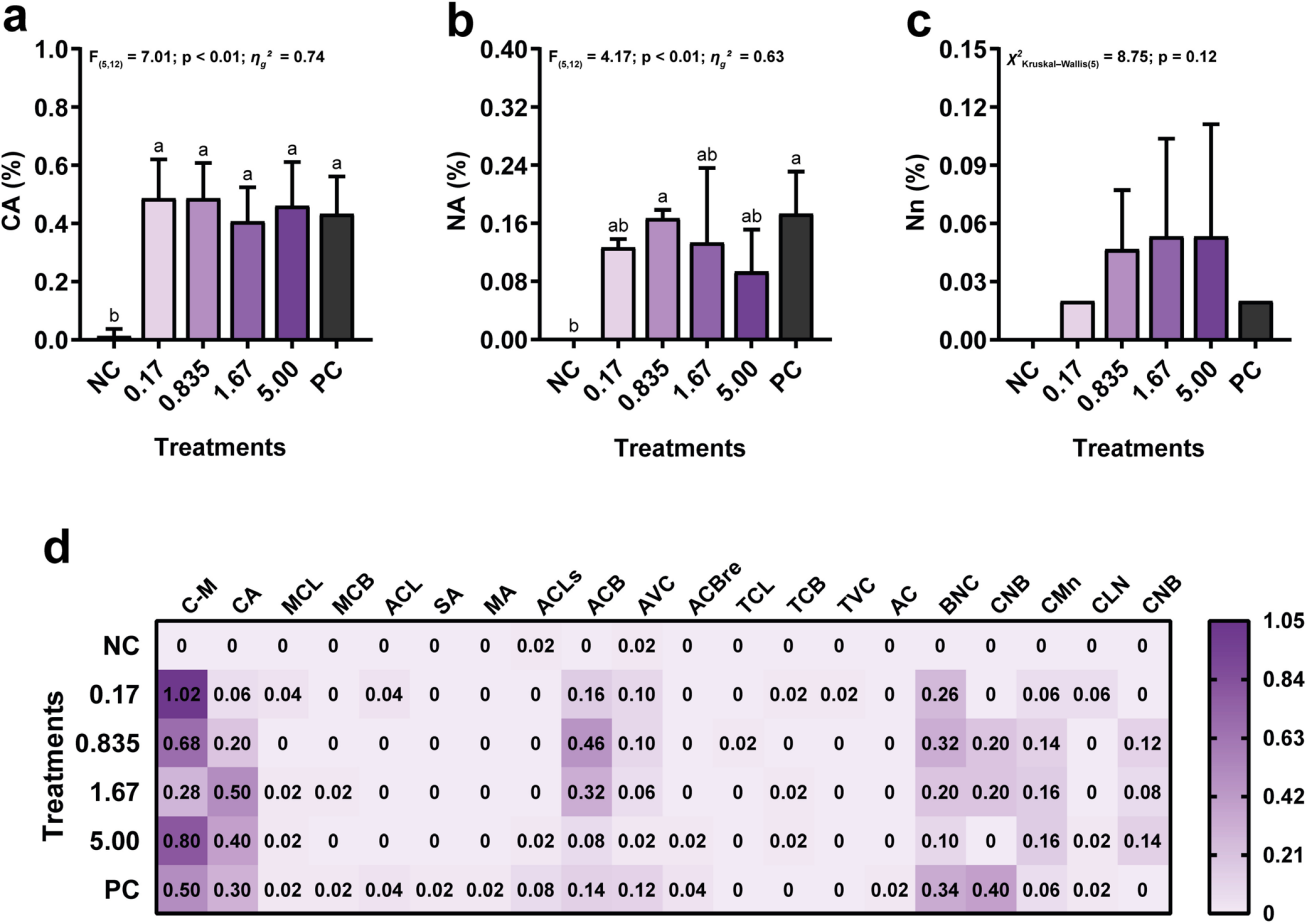
Cytogenetic alterations in *Allium cepa* roots exposed for 48 h to different concentrations of a fluopyram‐based pesticide and controls. Bars represent mean ± standard deviation. (a) Chromosomal aberration (CA) index. (b) Nuclear abnormality (NA) index. (c) Micronucleus (Mn) index. (d) Chromosomal aberration and nuclear abnormality frequencies, with darker colors indicating higher frequencies. NC = Negative control; PC = Positive control; C‐M = C‐mitosis; CA = Chromosomal adherence; MCL = Metaphase with chromosomal loss; MCB = Metaphase with chromosomal break; ACL = Anaphase with chromosomal lagging; SA = Star anaphase; MA = Multipolar anaphase; ACLs = Anaphase with chromosomal loss; ACB = Anaphase with chromosomal bridge; AVC = Anaphase with vagrant chromosomes; ACBre = Anaphase with chromosomal break; TCL = Telophase with chromosomal loss; TCB = Telophase with chromosomal bridge; TVC = Telophase with vagrant chromosomes; AC = Anucleated cell; BNC = Binucleated cell; CNB = Cell with nuclear bud; CMN = Cell with micronucleus; CLN = Cell with lobulated nucleus; CNB = Cell with nuclear bridge. Different letters indicate significant differences according to Tukey's post hoc test (*p* < 0.05); bars sharing the same letter do not differ significantly.

Despite these results, the micronucleus assay did not show significant differences among treatments (*χ* ^2^_Kruskal–Wallis(5)_ = 8.75; *p* = 0.12; Figure 3c), indicating that the fluopyram‐based pesticide did not induce micronucleus formation under the tested conditions. This finding is consistent with a predominantly aneugenic mode of action, likely involving disruption of spindle apparatus integrity rather than direct induction of DNA strand breaks.

Regarding the frequency of specific alterations, Figure 3d highlights the most prevalent chromosomal aberrations and nuclear abnormalities observed for each treatment and mitotic phase. C‐mitosis (C‐M) was the most frequent chromosomal aberration during the exposure, followed by chromosomal adherence (CA) and anaphase with chromosomal bridges (ACB). Among nuclear abnormalities, binucleated cells (BNC) predominated, followed by nuclear buds (CNB) and micronuclei (CMn). Representative photomicrographs of these alterations are presented in Figure 4 (chromosomal aberrations) and Figure 5 (nuclear abnormalities), providing visual confirmation of the cytogenotoxic effects induced by the fluopyram‐based pesticide.

**FIGURE 4:**
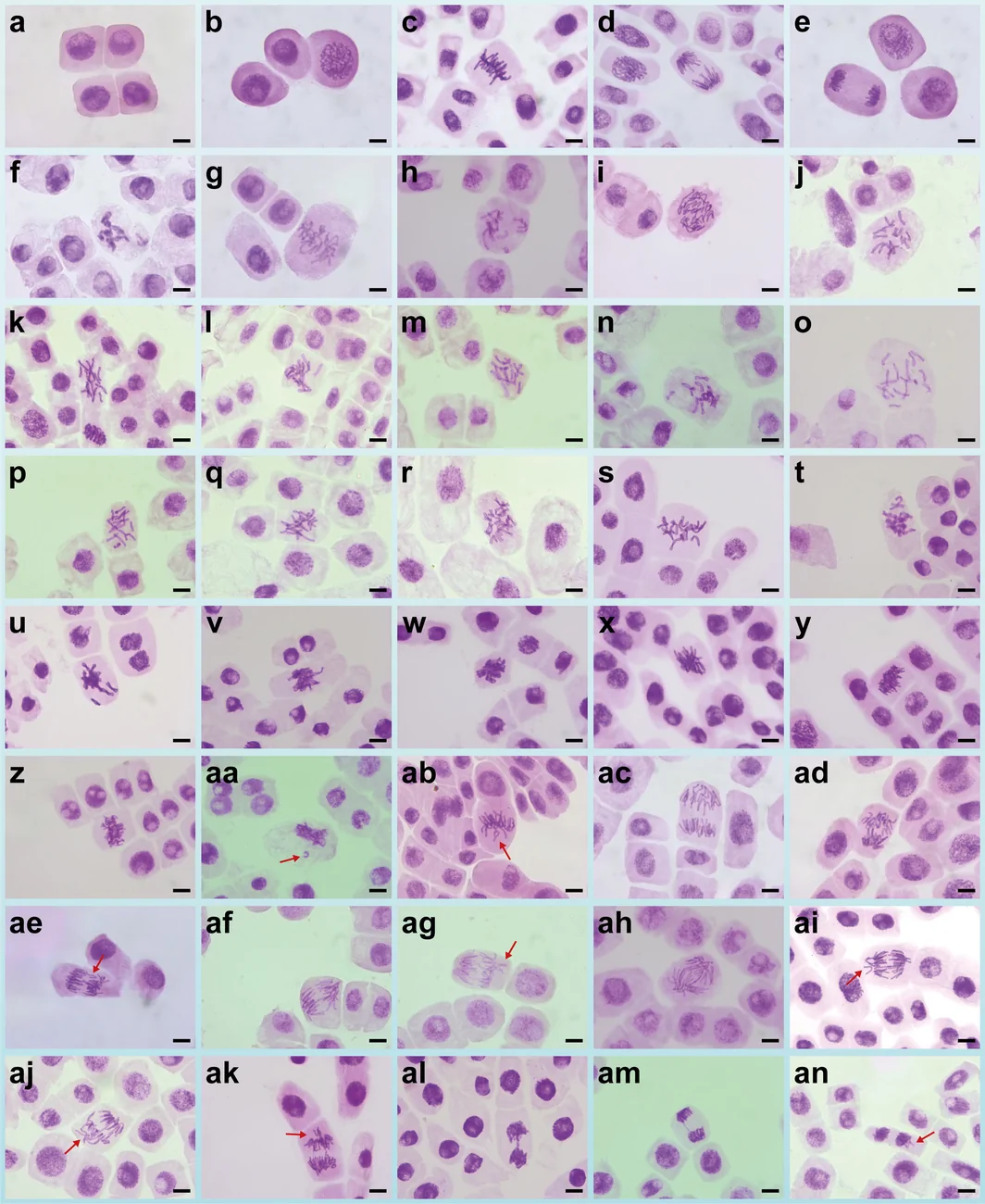
Representative photomicrographs of chromosomal aberrations observed in *Allium cepa* roots meristem cells after 48 h exposure to a fluopyram‐based pesticide and controls. (a) Normal interphase. (b) Normal prophase. (c) Normal metaphase. (d) Normal anaphase. (e) Normal telophase. (f–s) C‐mitosis. (t–u) Chromosomal adherence with loss. (v–z) Chromosomal adherence. (aa) Adherence with chromosomal breakage. (ab) Metaphase with chromosomal loss. (ac) Anaphase with chromosomal lagging. (ad, ag) Anaphase with bridges and chromosomal lagging. (ae) Anaphase with chromosomal loss. (af, ah) Anaphase with chromosomal bridges. (ai, aj) Anaphase with bridges and stray chromosomes. (ak) Anaphase with stray chromosomes. (al, am) Telophase with chromosomal bridges. (an) Telophase with stray chromosomes. Scale bar = 10 μm. Red arrows indicate representative aberrations.

**FIGURE 5:**
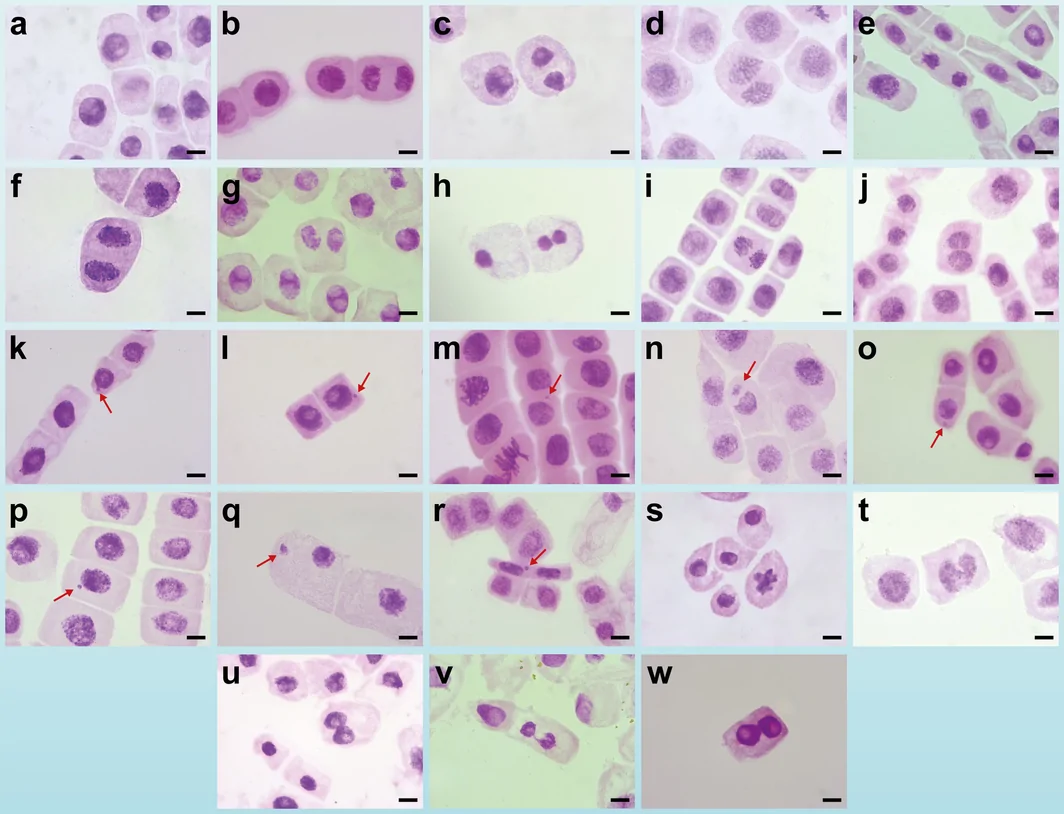
Representative photomicrographs of nuclear abnormalities observed in *Allium cepa* roots meristem cells after 48 h exposure to a fluopyram‐based pesticide. (a) Anucleated cell. (b–j) Binucleated cells. (k) Cell with nuclear bud. (l–r) Cells with micronucleus. (s–t) Cells with lobulated nucleus. (u–w) Interphase cells with nuclear bridge. Scale bar = 10 μm. Red arrows highlight micronuclei.

### 3.4 Comet Assay

The Comet assay performed on *A. cepa* root meristem cells showed that the fluopyram‐based pesticide did not induce marked DNA strand breaks at most of the tested concentrations. Analyses of tail length (Figure 6a), percentage of DNA in the tail (Figure 6b), and Olive Tail Moment (Figure 6c) revealed statistical differences among treatments. In addition, the representative nucleoids shown in Figure 6d–f visually support the progressive increase in DNA migration associated with higher levels of damage. However, only the highest concentration (5 mg L^−1^) resulted in a significant increase in DNA damage parameters compared with the negative control, whereas the remaining concentrations did not differ from the control. These results indicate that the fluopyram‐based pesticide exhibits low clastogenic activity, with detectable genotoxic effects occurring only at the highest exposure level.

**FIGURE 6:**
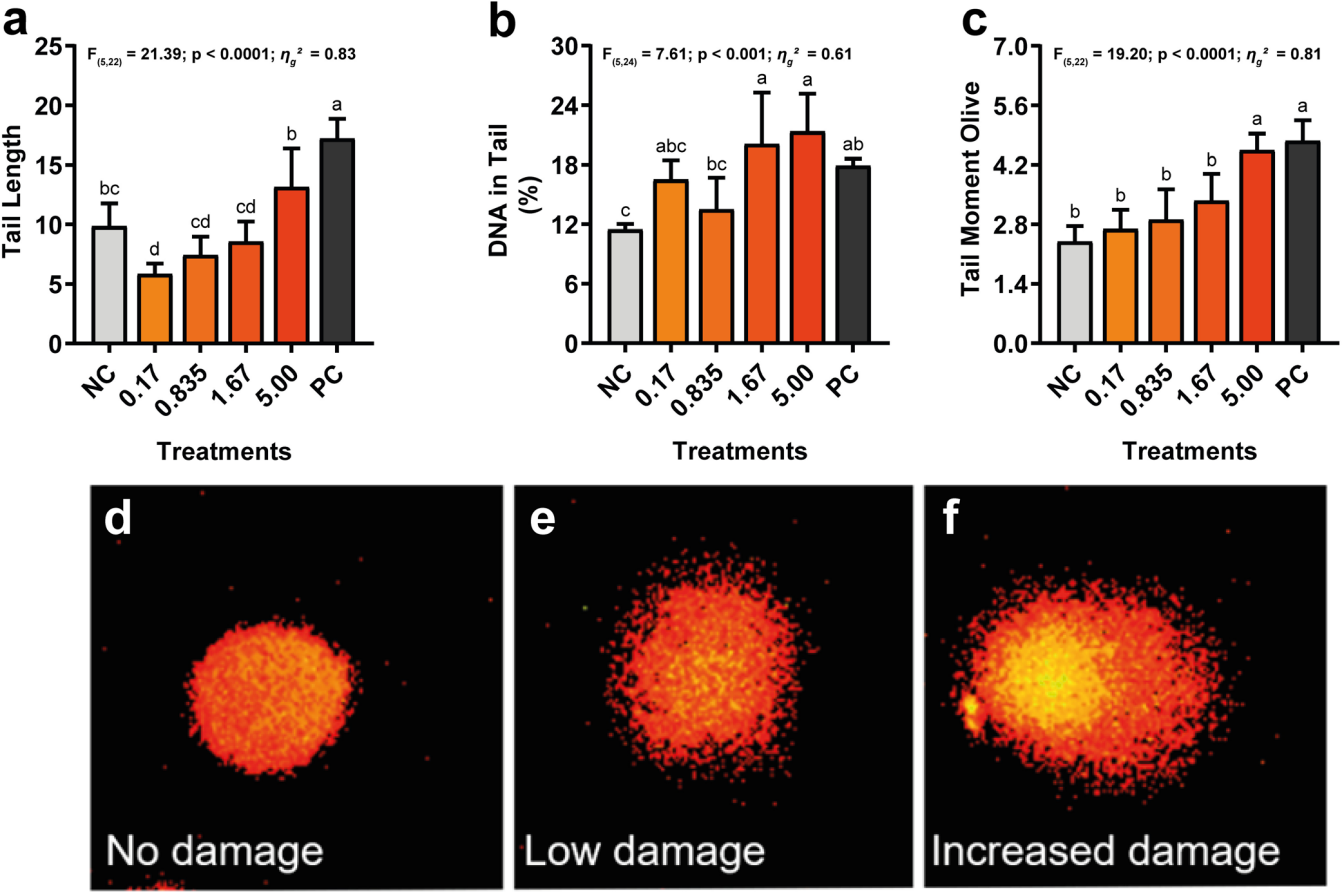
DNA damage parameters in *Allium cepa* roots exposed for 48 h to different concentrations of a fluopyram‐based pesticide and controls, assessed by the comet assay. Bars represent mean ± standard deviation. (a) Tail Length. (b) %DNA in Tail. (c) Olive Tail Moment. (d–f) Representative nucleoids illustrating increasing levels of DNA damage. CN = negative control; PC = positive control. Different letters indicate significant differences according to Tukey's post hoc test (*p* < 0.05); bars sharing the same letter do not differ significantly.

### 3.5 Molecular Docking and Pharmacophore Modeling

To further explore the hypothesis that fluopyram may exert its genotoxic effects through aneugenic mechanisms, potentially interfering with mitotic spindle function and promoting chromosomal missegregation, *in silico* molecular docking and pharmacophore modeling analyses were performed using tubulin as the target protein. Tubulin is a key structural component of the mitotic spindle, and previous studies have demonstrated that benzimidazole fungicides, such as benomyl, can bind to this protein and induce mitotic arrest by disrupting microtubule dynamics [35]. Based on this precedent, molecular docking simulations were conducted using a validated tubulin model to assess the potential interaction of fluopyram with the spindle apparatus.

The re‐docking of the co‐crystallized ligand (nocodazole) yielded RMSD values below 1.1 Å, validating the docking protocol [29]. Fluopyram fit well within the binding pocket, forming stabilizing interactions with residues LEU255 and ALA316 via hydrophobic interactions (3.7 and 4.0 Å, respectively), and establishing a hydrogen bond with TYR202 (2.5 Å) (Figure 7a,b).

**FIGURE 7:**
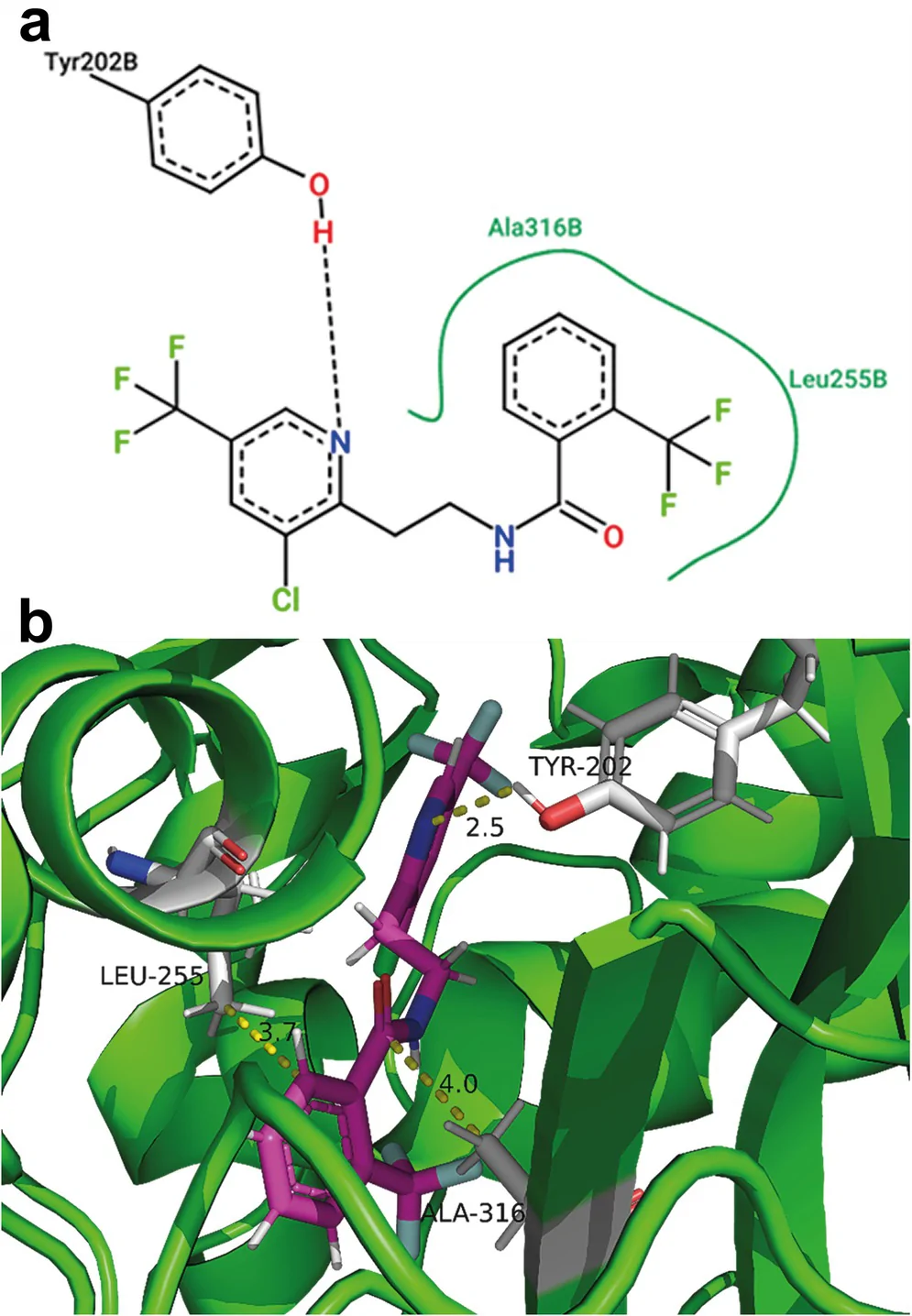
Molecular docking analysis of fluopyram in the active site of tubulin. This figure provides insights into the interaction patterns and binding conformations of fluopyram compared to a known inhibitor. (a) Two‐dimensional interaction diagram of fluopyram bound to tubulin in conformation 1, generated using PoseView (https://proteins.plus/ accessed on July 29, 2025). (b) Fluopyram docked in the ligand‐binding site of tubulin CYP17A1 in conformation 1, visualized with Pymol 3.1.3. Distance values are given in Å.

Pharmacophore modeling using LigandScout identified five key interaction features shared between fluopyram and known tubulin‐binding ligands: hydrogen bond acceptors (HBAs), hydrogen bond donors (HBDs), aromatic rings (ARs), and hydrophobic regions (HyPhos) (Figure 8). Notably, the fluorine atoms in fluopyram appear to contribute to binding affinity by participating in hydrogen bonding. Together, these structural features further support the potential of fluopyram to disrupt mitotic spindle function through direct interaction with tubulin.

**FIGURE 8:**
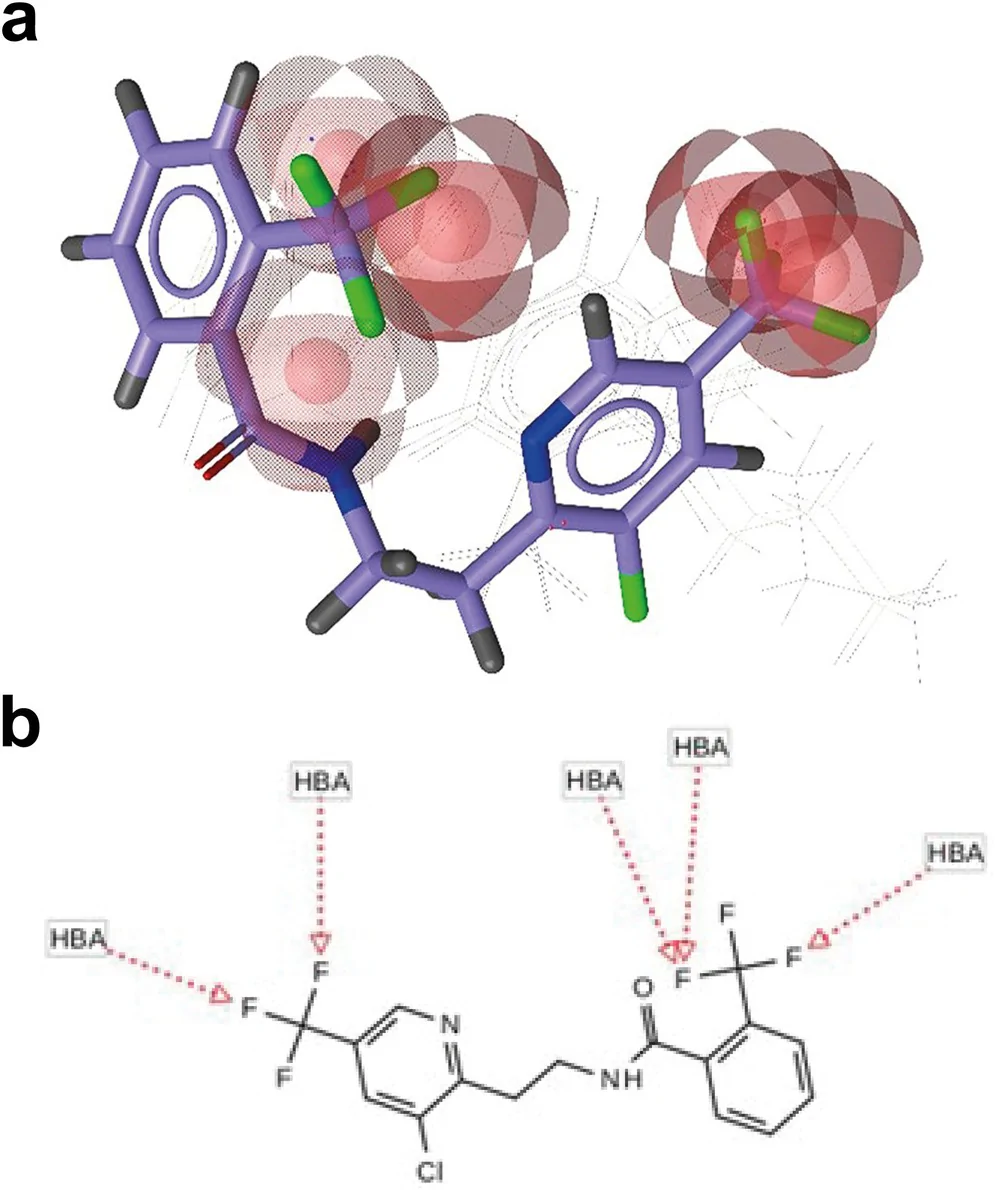
Pharmacophore modeling. (a) Three‐dimensional model of shared featured pharmacophore between fluopyram and the tubulin ligands. The pharmacophore features are color‐coded for hydrogen‐bond acceptors (red). The five compounds employed in this analysis were BDBM81744, BDBM81745, BDBM81746, BDBM81743, and BDBM81747. The shared spatial groups suggest a certain degree of similarity that reinforces the hypothesis that the fluopyram could have a similar activity to these substances, such as tubulin antagonists effects. (b) Individual pharmacophores of fluopyram shared with five ligands of tubulin. Hydrogen‐bond acceptor (HBA). These points where the pharmacophoric groups are located are potentially regions capable of interacting with amino acid residues in the active site of the tubulin.

## 4 Discussion

Assessing the toxicity of environmentally persistent pesticides is essential due to their potential long‐term effects on ecosystems and nontarget organisms. The *Allium cepa* assay remains a reliable model for detecting cytogenotoxicity, offering sensitivity, cost‐effectiveness, and good correlation with other test systems [14, 26]. In this study, a multiparametric approach encompassing cell viability, oxidative stress, chromosomal alterations, mitotic activity, and *in silico* molecular modeling was applied to evaluate the biological effects of a fluopyram‐based pesticide.

Lipid peroxidation and viability analyses provided complementary information. The absence of lipid peroxidation suggests the activation of adaptive mechanisms to preserve membrane integrity, possibly involving changes in lipid composition, regulation of membrane proteins, or the induction of detoxification pathways such as cytochrome P450 enzymes, which convert lipophilic compounds into more hydrophilic and excretable metabolites [36, 37]. Fluopyram, due to its high lipophilicity (log Pow = 3.3) [2], is likely metabolized through such pathways. The increased formazan levels observed at the highest concentration indicate enhanced mitochondrial activity and ATP production, which may support detoxification processes involving CYPs and glutathione [37, 38]. Mitochondrial adjustments to meet stress‐related energy demands [39] may explain the reduced Evans blue staining, as a higher proportion of viable cells remained metabolically active and capable of maintaining and repairing membrane integrity.

Mitotic index (MI) analysis revealed an increase at 0.835 mg mL^−1^ after 48 h of exposure, indicating stimulation of cell proliferation at this concentration. An elevated MI may be associated with uncontrolled cell proliferation and/or tumor formation [40]. This increase may be related to a shorter DNA repair time during the exposure period [41, 42]. According to the literature, MI reductions between −50% and − 78% indicate sublethal effects, whereas reductions beyond −78% are associated with lethality [40, 43, 44]. In our study, MI inhibition exceeded −50% at 5.00 mg mL^−1^, indicating considerable cytotoxicity.

Cell cycle analysis revealed a disproportionate accumulation of cells in prophase, accompanied by reduced frequencies in subsequent phases, except for telophase, which remained unchanged. This prophase arrest may be associated with CHFR‐mediated checkpoint activation, which delays entry into metaphase under mitotic stress [45, 46]. Similar findings have been reported in other *Allium cepa* studies, in which increased prophase frequencies were also linked to CHFR activation [19, 28, 41, 42, 47]. This type of cell cycle arrest is consistent with mitotic spindle disruption, a hypothesis further supported by the chromosomal aberrations observed in this study.

Cytogenetic analysis showed that the fluopyram‐based pesticide induced chromosomal aberrations at all evaluated concentrations compared with the negative control. Nuclear abnormalities also increased during exposure, although no significant increase in micronucleus frequency was detected. The comet assay revealed minimal DNA strand breaks, reinforcing the interpretation that aneugenic, rather than clastogenic, mechanisms predominate.

Pesticides can induce such effects through multiple pathways, including oxidative stress, direct interaction with DNA, and disruption of microtubules and histones, which compromise spindle formation and genetic material integrity [14]. As lipid peroxidation was not detected in our study, oxidative stress is unlikely to represent the primary pathway involved. Instead, the frequent occurrence of C‐mitosis, chromosomal adherence, lagging chromosomes, and vagrant chromosomes points to spindle dysfunction, consistent with inhibition of tubulin polymerization or depolymerization [24, 26, 41]. Partial spindle inhibition may generate lagging chromosomes, whereas complete inhibition results in C‐mitosis [48, 49, 50, 51]. Impaired microtubule depolymerization may further delay chromosome migration, leading to the formation of lagging or vagrant chromosomes [25, 52]. Together, these findings support the interpretation that fluopyram predominantly acts as an aneugenic agent by disrupting spindle fiber dynamics. Nevertheless, some clastogenic alterations were also observed, suggesting that direct interaction with DNA, such as intercalation or inhibition of DNA repair enzymes, may contribute secondarily to its genotoxicity effects [14].

*In silico* analyses strengthened the hypothesis of aneugenicity by demonstrating high‐affinity interactions between fluopyram and tubulin. This molecular interaction is likely to disrupt microtubule dynamics, thereby impairing chromosome segregation. Collectively, our findings demonstrate that fluopyram‐based pesticide exhibits cytotoxic and genotoxic effects in *A. cepa* , characterized by chromosomal aberrations and mitotic spindle disturbances. Given the strong correlation between the *A. cepa* assay and other biological models, including rat hepatocytes [53], bone marrow cells, and human leukocytes [54, 55], these results raise concerns regarding the potential effects of fluopyram on nontarget organisms, including humans. Despite being a modern fungicide, fluopyram induced effects comparable to those reported for older, highly toxic compounds such as mancozeb, procymidone, and imazalil [56, 57, 58]. This finding challenges assumptions about the safety of newer agrochemicals and underscores the need for comprehensive toxicological evaluations.

## 5 Conclusion

The fluopyram‐based pesticide significantly affected cell viability and cytogenotoxic parameters in *Allium cepa* root cells. These effects were concentration‐dependent and included reductions in the mitotic index, increased chromosomal aberrations, and elevated nuclear abnormalities. Although no oxidative stress was detected, the fluopyram‐based pesticide exhibited considerable genotoxic potential. Cytogenetic analysis indicated that the predominant mechanism of genotoxicity was aneugenic, as evidenced by the high frequency of c‐metaphases, chromosomal losses, and lagging chromosomes, which are hallmarks of mitotic spindle dysfunction. These findings suggest that the fluopyram‐based pesticide interferes with mitotic spindle formation, leading to chromosome missegregation and genomic instability.

This hypothesis was further supported by *in silico* molecular modeling, which revealed strong interactions between fluopyram and tubulin. These interactions may disrupt microtubule polymerization and dynamics, reinforcing the proposed aneugenic mechanism. Additionally, increased cell viability at higher concentrations may reflect a compensatory mitochondrial response aimed at sustaining energy production and supporting detoxification processes. Collectively, these results demonstrate that the fluopyram‐based pesticide exerts cytotoxic and genotoxic effects in a plant bioindicator model, primarily through aneugenic pathways involving microtubule disruption. Given the concordance between *A. cepa* assay outcomes and mammalian and human cell models, these findings raise important concerns regarding the safety of fluopyram in environmental and public health contexts.

## Author Contributions

**Carlos Filipe Camilo‐Cotrim:** conceptualization, methodology, software, validation, formal analysis, investigation, data curation, writing – original draft, writing – review and editing, visualization. **Natanael Alves da Silva:** investigation, methodology. **Gabriela Gomes Lima:** investigation, methodology. **Igor Rodrigues Vasconcelos:** cometa analysis and interpretation. **Luciana Souza Ondei:** resources, writing – review and editing. **Elisa Flávia Luiz Cardoso Bailão:** resources, writing – review and editing. **Antônio Sérgio Nakao de Aguiar:** *in silico* analysis and writing– review and editing. **Marcelino Benvindo‐Souza:** cometa analysis, writing – review and editing. **Leonardo Luiz Borges:** *in silico* analysis and writing – review and editing. **Virgínia Damin:** resources, writing – review and editing. **Luciane Madureira Almeida:** resources, writing – review and editing, supervision, project administration, funding acquisition.

## Funding

This work was supported by Coordenação de Aperfeiçoamento de Pessoal de Nível Superior (CAPES), Fundação de Amparo à Pesquisa do Estado de Goiás (03/2022, 09/2022), and Universidade Estadual de Goiás through its internal funding programs Pro‐Projetos and Pro‐Programas.

## Conflicts of Interest

The authors declare no conflicts of interest.

## Data Availability

The data that support the findings of this study are available from the corresponding author upon reasonable request.
